# Bis(2,3-dimethyl­anilinium) dihydrogen­diphosphate

**DOI:** 10.1107/S1600536810004149

**Published:** 2010-02-06

**Authors:** Houda Marouani, Lamia Elmi, Mohamed Rzaigui, Salem S. Al-Deyab

**Affiliations:** a Laboratoire de Chimie des Matériaux, Faculté des Sciences de Bizerte, 7021 Zarzouna Bizerte, Tunisia; b Petrochemical Research Chair, College of Science, King Saud University, Riyadh, Saudi Arabia

## Abstract

In the title compound, 2C_8_H_12_N^+^·H_2_P_2_O_7_^2−^, the complete dihydrogendiphosphate anion is generated by crystallographic twofold symmetry, with the bridging O atom lying on the rotation axis [P—O—P = 135.50 (9)°]. In the crystal, the 2,3-xylidinium cations are anchored between ribbons formed by the H_2_P_2_O_7_ entities. Crystal cohesion and stability are supported by electrostatic inter­actions which, together with N—H⋯O and O—H⋯O hydrogen bonds, build up a three-dimensional network.

## Related literature

For related structures, see: Akriche & Rzaigui (2000 ▶, 2001 ▶); Rayes *et al.* (2004 ▶); Aloui *et al.* (2006 ▶); Souissi *et al.* (2007 ▶). For a discussion on hydrogen bonding, see: Brown (1976 ▶); Blessing (1986 ▶). For tetra­hedral distortions, see: Baur (1974 ▶). For π–π inter­actions, see: Janiak (2000 ▶). 
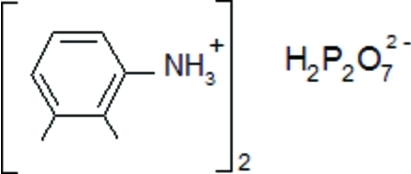


## Experimental

### 

#### Crystal data

2C_8_H_12_N^+^·H_2_P_2_O_7_^2−^
*M* *_r_* = 420.33
Monoclinic, 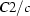

*a* = 32.9401 (10) Å
*b* = 4.5348 (10) Å
*c* = 15.560 (8) Å
β = 113.06 (4)°
*V* = 2138.6 (12) Å^3^
*Z* = 4
Ag *K*α radiation
λ = 0.56087 Å
μ = 0.13 mm^−1^
*T* = 293 K
0.50 × 0.45 × 0.25 mm

#### Data collection

Enraf–Nonius CAD-4 diffractometer
3944 measured reflections
3815 independent reflections
2891 reflections with *I* > 2σ(*I*)
*R*_int_ = 0.011
2 standard reflections every 120 min  intensity decay: 1%

#### Refinement

*R*[*F* ^2^ > 2σ(*F* ^2^)] = 0.040
*wR*(*F* ^2^) = 0.138
*S* = 1.11
3815 reflections
127 parameters
H-atom parameters constrained
Δρ_max_ = 0.44 e Å^−3^
Δρ_min_ = −0.22 e Å^−3^

### 

Data collection: *CAD-4 EXPRESS* (Enraf–Nonius, 1994 ▶); cell refinement: *CAD-4 EXPRESS*; data reduction: *XCAD4* (Harms & Wocadlo, 1995 ▶); program(s) used to solve structure: *SHELXS97* (Sheldrick, 2008 ▶); program(s) used to refine structure: *SHELXL97* (Sheldrick, 2008 ▶); molecular graphics: *ORTEP-3 for Windows* (Farrugia, 1997 ▶); software used to prepare material for publication: *WinGX* (Farrugia, 1999 ▶).

## Supplementary Material

Crystal structure: contains datablocks I, global. DOI: 10.1107/S1600536810004149/hb5321sup1.cif

Structure factors: contains datablocks I. DOI: 10.1107/S1600536810004149/hb5321Isup2.hkl

Additional supplementary materials:  crystallographic information; 3D view; checkCIF report

## Figures and Tables

**Table 1:** Hydrogen-bond geometry (Å, °)

*D*—H⋯*A*	*D*—H	H⋯*A*	*D*⋯*A*	*D*—H⋯*A*
O3—H3⋯O1^i^	0.82	1.70	2.5088 (15)	167
N1—H1*A*⋯O4	0.89	1.81	2.6733 (15)	163
N1—H1*B*⋯O4^i^	0.89	1.99	2.8175 (16)	154
N1—H1*C*⋯O1^ii^	0.89	1.85	2.732 (2)	172

Symmetry codes: (i) 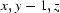; (ii) 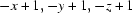.

**Fig. 1.**
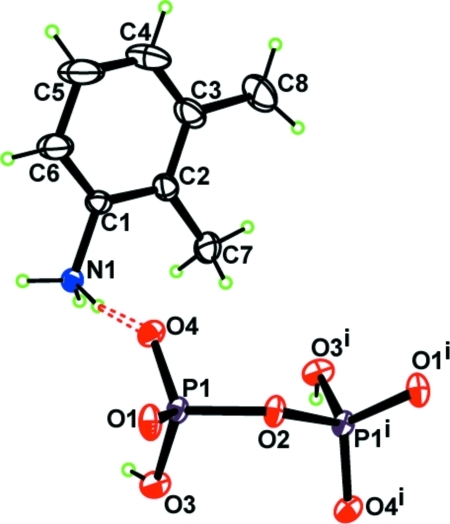
View of (I) with displacement ellipsoids drawn at the 30% probability level. H atoms are represented as spheres of arbitrary radius. Hydrogen bonds are represented as dashed lines [Symmetry code: (i) (-x+1, -y+1, -z+1)] x,

**Fig. 2.**
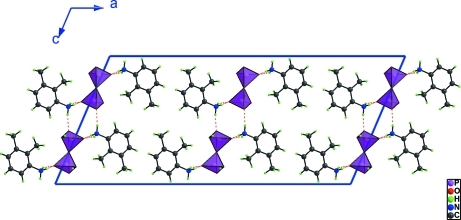
Projection of (I) along the b axis.

## supplementary crystallographic information

### Comment

The design and synthesis of inorganic-organic hybrid materials have been great interest due to their unique opportunity to combine the remarkable features of organic materials with those of inorganic compounds. In particular the family of material which combine phosphate anions with organic molecules have received much attention in recent years due to their technological interest in several areas such as biomolecular sciences, catalysts and optics.

In order to research new materials of this kind and to investigate the influence of hydrogen bonds on the chemical and structural features, we report here synthesis and crystal structure of a new organic diphosphate, [2,3-(CH_3_)_2_C_6_H_3_NH_3_]_2_H_2_P_2_O_7_ , (I).

Chemical formula of the title compound is built up from (H_2_P_2_O_7_)^2-^ anion and two organic 2,3-xylidinium cations. Its geometrical configuration is depicted in the figure 1. The half of this formula constitutes the asymmetric unit in the atomic arrangement. This latter is characterized by the existence of infinite ribbons built by H_2_P_2_O_7_^2-^ anions. The phosphoric chains, extending along the b-direction, are located around planes perpendicular to the a-axis at x = 0 and x = 1/2 (Fig.2). The bridging oxygen atom of H_2_P_2_O_7_^2-^ anion is located on the twofold axis, thus this anion has a binary internal symmetry and so is built by only one independent PO_4_ tetrahedron. The H_2_P_2_O_7_ entities are connected between themselves by strong hydrogen bonds O(1)···O(2) = 1.505 (1) Å (Table 1) , to form infinite ribbons in the b-direction. These ribbons are linked via N—H···O hydrogen bonds generating a three-dimensional network.

The average values for P—O distances and angles are quite similar to those measured in diphosphate anions with the same internal symmetry (Akriche, *et al.*, 2000,2001). Nevertheless, the calculated average values of the distortion indice (Baur, 1974) corresponding to the different angles and distances in the independent PO_4_ tetrahedron, DI(PO) = 0.0236, DI(OPO) = 0.0312 and ID(OO) = 0.0100, show a distortion of P—O distances compared to O—O distances. The PO_4_ tetrahedron is thus described by a regular oxygen atom arrangement with the phosphorus atom slightly shifted from the gravity center of PO_4_.

In this atomic arrangement, the 2,3-xylidine molecule is protonated for neutralize the negative charge of the anionic part. The 2,3-xylidinium cations are organized in a similar direction. They create intermolecular van der Waals interactions between them and establish strong hydrogen bonds (Blessing, 1986); (Brown, 1976) with oxygen atoms of the anionic layers.

Each organic entity is bounded to three different H_2_P_2_O_7_^2-^ groups through three N—H···O hydrogen bonds. It exhibits a regular spatial configuration with usual interatomic distances C—C, C—N and angles C—C—C, C—C—N, spreading within the respective ranges of 1.370 (4)-1.510 (3) Å and 116.9 (1)-123.2 (1)°. These values are similar to those obtained in other organic phosphates associated to the same organic groups (Rayes, *et al.*, 2004); (Aloui, *et al.*, ,2006). The aromatic ring of the protonated amine is planar, with a mean plane deviation of 0.0015 Å . The interplanar distance between the aryl rings of the organic cation is in the vicinity of 4.54 Å , which is significantly longer than 3.80 Å for the π–π interaction (Janiak, 2000). However, it should be noticed that the same organic groups display *p*-p interaction in [2,3-(CH_3_)_2_C_6_H_3_NH_3_]_4_P_4_O_12_.2H_2_O (Aloui, *et al.*, 2006)and in [2,3-(CH_3_)_2_C_6_H_3_NH_3_]_4_HP_3_O_10_.2H_2_O (Souissi,*and al.*, 2007) with an interplanar distance of 3.78 Å and 3.38 Å , respectively.

### Experimental

The diphosphoric acid was prepared by passing a concentrated solution of Na_4_P_2_O_7_.10H_2_O (4 mmol) through an ion-exchange resin (Amberlite IR 120) in its H-State. The diphosphoric acid was then neutralized with an ethanol solution (10 ml) of 2,3-xylidine (8 mmol) by mixing them at 273 K in stoichiometric ratio 1:2. The resulting solution was slowly evaporated at room temperature until the formation of pink prisms of (I), which were stable under normal condition of temperature and humidity.

### Refinement

(type here to add refinement details)

### Crystal data

**Table:**

2C_8_H_12_N^+^·H_2_P_2_O_7_^2^^−^	*F*(000) = 888
*M**_r_* = 420.33	*D*_x_ = 1.305 Mg m^−^^3^
Monoclinic, *C*2/*c*	Ag *K*α radiation, λ = 0.56087 Å
Hall symbol: -C 2yc	Cell parameters from 25 reflections
*a* = 32.9401 (10) Å	θ = 8–10°
*b* = 4.5348 (10) Å	µ = 0.13 mm^−^^1^
*c* = 15.560 (8) Å	*T* = 293 K
β = 113.06 (4)°	Prism, pink
*V* = 2138.6 (12) Å^3^	0.50 × 0.45 × 0.25 mm
*Z* = 4	

### Data collection

**Table:**

Enraf–Nonius CAD-4 diffractometer	*R*_int_ = 0.011
Radiation source: Enraf Nonius FR590	θ_max_ = 25.0°, θ_min_ = 2.1°
graphite	*h* = −49→45
non–profiled ω scans	*k* = 0→6
3944 measured reflections	*l* = 0→23
3815 independent reflections	2 standard reflections every 120 min
2891 reflections with *I* > 2σ(*I*)	intensity decay: 1%

### Refinement

**Table:**

Refinement on *F*^2^	Primary atom site location: structure-invariant direct methods
Least-squares matrix: full	Secondary atom site location: difference Fourier map
*R*[*F*^2^ > 2σ(*F*^2^)] = 0.040	Hydrogen site location: inferred from neighbouring sites
*wR*(*F*^2^) = 0.138	H-atom parameters constrained
*S* = 1.11	*w* = 1/[σ^2^(*F*_o_^2^) + (0.0801*P*)^2^ + 0.3731*P*] where *P* = (*F*_o_^2^ + 2*F*_c_^2^)/3
3815 reflections	(Δ/σ)_max_ = 0.001
127 parameters	Δρ_max_ = 0.44 e Å^−^^3^
0 restraints	Δρ_min_ = −0.22 e Å^−^^3^

### Special details

**Table:**

Geometry. All esds (except the esd in the dihedral angle between two l.s. planes) are estimated using the full covariance matrix. The cell esds are taken into account individually in the estimation of esds in distances, angles and torsion angles; correlations between esds in cell parameters are only used when they are defined by crystal symmetry. An approximate (isotropic) treatment of cell esds is used for estimating esds involving l.s. planes.
Refinement. Refinement of *F*^2^ against ALL reflections. The weighted *R*-factor wR and goodness of fit *S* are based on *F*^2^, conventional *R*-factors *R* are based on *F*, with *F* set to zero for negative *F*^2^. The threshold expression of *F*^2^ > σ(*F*^2^) is used only for calculating *R*-factors(gt) etc. and is not relevant to the choice of reflections for refinement. *R*-factors based on *F*^2^ are statistically about twice as large as those based on *F*, and *R*- factors based on ALL data will be even larger.

### Fractional atomic coordinates and isotropic or equivalent isotropic displacement parameters (Å^2^)

**Table:**

	*x*	*y*	*z*	*U*_iso_*/*U*_eq_	
P1	0.510805 (12)	0.68375 (7)	0.35144 (2)	0.03189 (11)	
O1	0.53139 (4)	0.9396 (2)	0.41487 (7)	0.0424 (3)	
O2	0.5000	0.8169 (3)	0.2500	0.0384 (3)	
O3	0.54560 (4)	0.4412 (2)	0.36452 (9)	0.0470 (3)	
H3	0.5369	0.2852	0.3779	0.071*	
O4	0.46936 (4)	0.5703 (2)	0.35653 (8)	0.0424 (2)	
N1	0.43242 (4)	0.0895 (3)	0.39453 (8)	0.0350 (2)	
H1A	0.4485	0.2236	0.3802	0.052*	
H1B	0.4353	−0.0845	0.3711	0.052*	
H1C	0.4417	0.0755	0.4563	0.052*	
C1	0.38624 (5)	0.1778 (4)	0.35532 (12)	0.0445 (3)	
C2	0.36165 (6)	0.1438 (4)	0.26065 (13)	0.0540 (4)	
C3	0.31819 (7)	0.2505 (7)	0.22611 (18)	0.0764 (7)	
C4	0.30194 (8)	0.3801 (8)	0.2858 (3)	0.1040 (11)	
H4	0.2730	0.4483	0.2622	0.125*	
C5	0.32707 (9)	0.4116 (9)	0.3788 (3)	0.1165 (13)	
H5	0.3154	0.5022	0.4177	0.140*	
C6	0.36968 (7)	0.3087 (6)	0.41443 (18)	0.0796 (7)	
H6	0.3871	0.3273	0.4778	0.095*	
C7	0.38165 (8)	0.0022 (7)	0.19904 (14)	0.0804 (7)	
H7A	0.4052	0.1237	0.1972	0.121*	
H7B	0.3595	−0.0192	0.1370	0.121*	
H7C	0.3931	−0.1885	0.2235	0.121*	
C8	0.28886 (9)	0.2238 (11)	0.1235 (2)	0.1297 (14)	
H8A	0.2637	0.3508	0.1092	0.195*	
H8B	0.2791	0.0234	0.1096	0.195*	
H8C	0.3051	0.2803	0.0867	0.195*	

### Atomic displacement parameters (Å^2^)

**Table:**

	*U*^11^	*U*^22^	*U*^33^	*U*^12^	*U*^13^	*U*^23^
P1	0.04612 (19)	0.02030 (15)	0.03291 (17)	0.00149 (12)	0.01944 (14)	0.00289 (11)
O1	0.0698 (7)	0.0241 (4)	0.0301 (5)	−0.0029 (4)	0.0160 (4)	0.0017 (4)
O2	0.0632 (9)	0.0255 (6)	0.0304 (6)	0.000	0.0226 (6)	0.000
O3	0.0532 (6)	0.0243 (4)	0.0715 (8)	0.0059 (4)	0.0330 (6)	0.0104 (5)
O4	0.0523 (6)	0.0313 (5)	0.0537 (6)	0.0016 (4)	0.0316 (5)	0.0055 (4)
N1	0.0379 (5)	0.0330 (5)	0.0349 (5)	−0.0003 (4)	0.0152 (4)	0.0025 (4)
C1	0.0365 (6)	0.0454 (8)	0.0512 (8)	−0.0012 (6)	0.0169 (6)	0.0025 (7)
C2	0.0410 (7)	0.0636 (11)	0.0511 (9)	−0.0091 (7)	0.0113 (7)	0.0096 (8)
C3	0.0417 (9)	0.0942 (16)	0.0760 (14)	−0.0044 (11)	0.0043 (9)	0.0159 (14)
C4	0.0411 (10)	0.124 (2)	0.135 (3)	0.0155 (13)	0.0218 (14)	−0.008 (2)
C5	0.0570 (13)	0.169 (3)	0.123 (3)	0.0279 (18)	0.0343 (16)	−0.037 (2)
C6	0.0507 (10)	0.112 (2)	0.0743 (14)	0.0127 (12)	0.0227 (10)	−0.0250 (14)
C7	0.0648 (12)	0.129 (2)	0.0413 (9)	−0.0042 (14)	0.0146 (8)	−0.0027 (12)
C8	0.0561 (13)	0.213 (4)	0.084 (2)	−0.003 (2)	−0.0123 (13)	0.030 (3)

### Geometric parameters (Å, °)

**Table:**

P1—O4	1.4900 (11)	C3—C4	1.372 (4)
P1—O1	1.5020 (11)	C3—C8	1.513 (3)
P1—O3	1.5433 (11)	C4—C5	1.364 (5)
P1—O2	1.5945 (10)	C4—H4	0.9300
O2—P1^i^	1.5944 (10)	C5—C6	1.373 (3)
O3—H3	0.8200	C5—H5	0.9300
N1—C1	1.4559 (19)	C6—H6	0.9300
N1—H1A	0.8900	C7—H7A	0.9600
N1—H1B	0.8900	C7—H7B	0.9600
N1—H1C	0.8900	C7—H7C	0.9600
C1—C6	1.374 (3)	C8—H8A	0.9600
C1—C2	1.384 (2)	C8—H8B	0.9600
C2—C3	1.404 (3)	C8—H8C	0.9600
C2—C7	1.503 (3)		
			
O4—P1—O1	114.78 (7)	C2—C3—C8	120.7 (3)
O4—P1—O3	113.29 (6)	C5—C4—C3	121.8 (2)
O1—P1—O3	110.06 (7)	C5—C4—H4	119.1
O4—P1—O2	109.24 (6)	C3—C4—H4	119.1
O1—P1—O2	103.08 (6)	C4—C5—C6	119.5 (3)
O3—P1—O2	105.47 (6)	C4—C5—H5	120.2
P1^i^—O2—P1	135.50 (9)	C6—C5—H5	120.2
P1—O3—H3	109.5	C5—C6—C1	119.2 (3)
C1—N1—H1A	109.5	C5—C6—H6	120.4
C1—N1—H1B	109.5	C1—C6—H6	120.4
H1A—N1—H1B	109.5	C2—C7—H7A	109.5
C1—N1—H1C	109.5	C2—C7—H7B	109.5
H1A—N1—H1C	109.5	H7A—C7—H7B	109.5
H1B—N1—H1C	109.5	C2—C7—H7C	109.5
C6—C1—C2	122.71 (18)	H7A—C7—H7C	109.5
C6—C1—N1	117.50 (17)	H7B—C7—H7C	109.5
C2—C1—N1	119.68 (16)	C3—C8—H8A	109.5
C1—C2—C3	116.9 (2)	C3—C8—H8B	109.5
C1—C2—C7	120.43 (17)	H8A—C8—H8B	109.5
C3—C2—C7	122.6 (2)	C3—C8—H8C	109.5
C4—C3—C2	119.9 (2)	H8A—C8—H8C	109.5
C4—C3—C8	119.4 (3)	H8B—C8—H8C	109.5

Symmetry codes: (i) −*x*+1, *y*, −*z*+1/2.

### Hydrogen-bond geometry (Å, °)

**Table:**

*D*—H···*A*	*D*—H	H···*A*	*D*···*A*	*D*—H···*A*
O3—H3···O1^ii^	0.82	1.70	2.5088 (15)	167
N1—H1A···O4	0.89	1.81	2.6733 (15)	163
N1—H1B···O4^ii^	0.89	1.99	2.8175 (16)	154
N1—H1C···O1^iii^	0.89	1.85	2.732 (2)	172

Symmetry codes: (ii) *x*, *y*−1, *z*; (iii) −*x*+1, −*y*+1, −*z*+1.
